# Longitudinally extensive transverse myelopathy in spinal cord infarction

**DOI:** 10.1002/ccr3.6671

**Published:** 2022-12-26

**Authors:** Yi Rong Chiew, Jeanne May May Tan

**Affiliations:** ^1^ Department of Neurology National Neuroscience Institute Singapore Singapore

**Keywords:** inflammatory myelitis, longitudinally extensive transverse myelopathy, neuromyelitis optica, spinal cord infarction

## Abstract

Spinal cord infarction may present as longitudinally extensive myelopathy, similar to inflammatory myelitis such as neuromyelitis optica. Magnetic resonance imaging features such as diffusion‐weighted imaging/apparent diffusion coefficient showing restricted diffusion and lack of contrast enhancement are helpful in the diagnosis of spinal cord infarction and differentiating them from inflammatory myelitis.

## Abstract 2

Spinal cord infarction may present with longitudinally extensive myelopathy on MRI, which is similar to inflammatory myelitis such as neuromyelitis optica. Diffusion‐weighted imaging and apparent diffusion coefficient showing restricted diffusion, with lack of contrast enhancement on spinal MRI, are helpful in differentiating between spinal cord infarction and neuromyelitis optica.

## 1 CASE PRESENTATION

**FIGURE 1:**
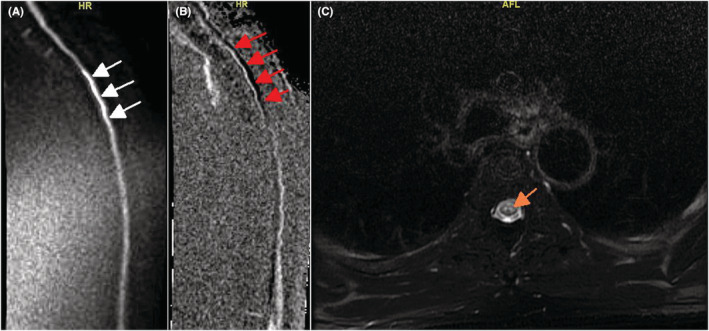
Sagittal plane of MRI spine DWI sequence (A, white arrows) and ADC (B, red arrows) showing restricted diffusion suggesting acute spinal cord infarction from T3 to T5 levels. Axial plane T2‐weighted sequence of MRI spine showing the Owl's Eyes Sign (C, orange arrow)

A previously healthy, 58‐year‐old man developed sudden onset acute weakness and numbness of the lower limbs. His examination revealed bilateral lower limbs weakness, decreased pain, and temperature sensation with a sensory level at T5 level. His proprioception and vibration senses were normal. His Babinski responses were abnormal bilaterally. A contrast‐enhanced thoracic spine MRI with DWI and ADC sequences was ordered, and the result was consistent with a spinal cord infarction (Figure 1). A lumbar puncture was performed and was normal. Many patients with spinal cord infarction (SCI) had DWI/ADC‐restricted diffusion, without contrast enhancement, and T2 hyperintensity showing Owl Eyes sign in their MRI, indicating disruption of the anterior spinal artery.^1^ Similar to neuromyelitis optica (NMO), SCI may have longitudinally extensive involvement of the spine,^2^ however, in SCI, contrast enhancement is absent due to disrupted blood flow, whereas NMO lesions typically show enhancement as a result of inflammation with intact blood flow. In contrast to SCI, NMO lesions usually affect areas with good collateral circulation.^2^ In conclusion, DWI/ADC sequences and contrast administration should be performed in spinal MRI, especially in patients highly suspected of SCI, to allow prompt diagnosis and to differentiate spinal cord infarction from NMO.

## AUTHOR CONTRIBUTIONS

Yi Rong Chiew contributed to the planning of study, conceptualization, acquisition, and analysis of data and writing (original draft, review, and editing) of the manuscript. Tan May May Jeanne (co‐first author) contributed to the planning of study, conceptualization, acquisition, and analysis of data and writing (original draft, review, and editing) of the manuscript.

## CONFLICT OF INTEREST

There is no conflict of interest.

## ETHICAL APPROVAL

Not applicable.

## CONSENT

Informed consent had been obtained from the patient for the publication of this case report and its accompanying investigation results.

## Data Availability

We documented the patient's data reported in the article. We will share the de‐identified data on reasonable request. To request the data, please contact the corresponding author.
